# Reasons for unmet need for family planning, with attention to the measurement of fertility preferences: protocol for a multi-site cohort study

**DOI:** 10.1186/s12978-016-0268-z

**Published:** 2017-02-09

**Authors:** Kazuyo Machiyama, John B. Casterline, Joyce N. Mumah, Fauzia Akhter Huda, Francis Obare, George Odwe, Caroline W. Kabiru, Sharifa Yeasmin, John Cleland

**Affiliations:** 1 Faculty of Epidemiology and Population Health, London School of Hygiene and Tropical Medicine, Keppel Street, London, WC1E 7HT UK; 2 Institute for Population Research, Ohio State University, Columbus, USA; 3 African Population and Health Research Center, Nairobi, Kenya; 4 icddr,b, Dhaka, Bangladesh; 5 Population Council, Nairobi, Kenya

**Keywords:** Family planning, Study protocol, Kenya, Bangladesh, Unmet need for family planning, Fertility, Fertility preferences, Measurement

## Abstract

**Background:**

Unmet need for family planning points to the gap between women’s reproductive desire to avoid pregnancy and contraceptive behaviour. An estimated 222 million women in low- and middle-income countries have unmet need for modern contraception. Despite its prevalence, there has been little rigorous research during the past fifteen years on reasons for this widespread failure to implement childbearing desires in contraceptive practice. There is demographic survey data on women’s self-reported reasons for non-use, but these data provide limited insight on the full set of possible obstacles to use, and one may doubt the meaningfulness of explanations provided by non-users alone. To rectify this evidence gap, this study will gather extensive information on women’s perceptions of contraception (generic and method-specific) and their past contraceptive experience, and it will allow for more complexity in fertility preferences than is standard in demographic surveys.

**Methods:**

A multi-site cohort study will be conducted in urban Kenya, rural Kenya, and rural Bangladesh. In each setting trained fieldworkers will recruit and interview 2600 women, with participants re-interviewed at 12 and 18 months. Data will be collected using a questionnaire whose development was informed by a review of existing literature and instruments from past studies in both developed and developing countries. Dozens of experts in the field were consulted as the instrument was developed. The questionnaire has three main components: a sub-set of Demographic and Health Survey items measuring socio-demographic characteristics, reproductive history, and sexual activity; additional questions on prospective and retrospective fertility preferences designed to capture ambivalence and uncertainty; and two large blocks of items on (i) generic concerns about contraception and (ii) method-specific attributes. The method-specific items encompass eight modern and traditional methods.

**Discussion:**

Policy and programmes intended to reduce unmet need for contraception in developing countries should be informed by clear understanding of the causes of this phenomenon to better reflect the population needs and to more effectively target planning and investments. To this end, this study will field an innovative instrument in Kenya and Bangladesh. The information to be collected will support a rigorous assessment of reasons for unmet need for family planning.

## Plain English summary

In low- and middle-income countries, about 220 million women who want to avoid future childbirth are not using modern methods or techniques to prevent pregnancy. However, reasons for non-use of contraception  are not well understood. There have been major drawbacks with the existing approaches, especially the failure to distinguish contraceptives users from non-users mainly because of the specific focus on non-users alone. This study aims to fill this important gap in scientific evidence through intensive assessment of women’s perceptions of contraception, both in general and with respect to specific methods, and their past experience with contraception. The study also pays special attention to understanding the degree to which women want to avoid future pregnancies.

This protocol describes data collection to be carried out in three sites: two sites in Kenya (rural and urban), and one site in rural Bangladesh. The survey tool consists of three main components: 1) socio-demographic characteristics, reproductive history, sexual activity; 2) detailed inquiry about the desire to have another child; and 3) innovative blocks of questions on contraceptive perceptions and experience, including generic attitudes towards pregnancy-prevention, and method-specific perceptions and past contraceptive experiences. In each setting the study will recruit a cohort of 2600 women in union aged 15–39 at baseline, and the women will be re-interviewed at 12 and 18 months.

This rigorous assessment of reasons for unmet need for family planning will contribute knowledge to the evidence base which can inform policy and programmes intended to reduce unmet need in low- and middle-income countries.

## Background

Reducing unmet need for family planning has been a central aim for reproductive health policy, programmes and research for decades. Unmet need for family planning refers to a discrepancy between expressed fertility preferences and practice of contraception – i.e., the failure to translate a stated desire to avoid pregnancy into pregnancy-prevention behaviour. Women who indicate that they do not want another child or would like to postpone the next birth for at least two years but are not using any method of contraceptive are classified as having an unmet need for family planning. Despite recent progress in decreasing its prevalence, it is estimated that 222 million women in low- and middle-income countries have an unmet need for modern contraception [1].

Global and national pronouncements and policies often assume that the cause of unmet need is women’s inability to access family planning services. For instance, the stated goal of the FP2020 Initiative is to provide an additional 120 million women with access to contraception by 2020 [2]. However, there is ample evidence from research on family planning over the decades, not to mention the abundant anecdotal testimony of field workers that the obstacles to contraception go well beyond access to services that are of good quality and affordable [3]. Non-access barriers include fear of health side effects, normative acceptability (including religious concerns), social acceptability (including the important matter of the partner’s approval), and various possible informational and other cultural factors [4]. Uncertainty and ambivalence about the desire to avoid pregnancy can also figure in, as can perceptions of the actual risk of becoming pregnant [5, 6].

The need to adopt a broad understanding of the obstacles to contraception emerged from research conducted in the 1980s and 1990s and has been reinforced by more recent research. This research reveals the large weight of concerns about social and cultural factors as compared to objective features of the service environment, e.g., distance or travel time to family planning service outlets. A study that assessed objective distance to family planning services and contraceptive use suggested that physical access is likely to affect contraceptive uptake largely through women’s knowledge of a supply source [7]. A more recent study in Indonesia showed only a moderate impact of government contraceptive investments on contraceptive use [8]. Social, cultural and moral acceptability and informational barriers are also significant contributors to women’s non-use of contraception, particularly in settings with low prevalence of contraception [9–11].

The existing research literature also suggests that understanding the causes of unmet need for family planning requires better appreciation of the complexity of fertility desires. Simply stated, some women who express a wish to avoid pregnancy may be uncertain or ambivalent about this preference, or it may be weakly held, and therefore they possess insufficient motivation to take the steps required to practice contraception [5]. This in turn raises questions about the adequacy of standard survey measures of fertility desires. Major demographic survey programmes (e.g., the Demographic and Health Surveys [DHS], the Multiple Indicator Cluster Surveys [MICS]) have relied on three items: an item asking for the woman’s ideal number of children (ideal family size); an item asking about her desire to have another child, and the desired timing of the next birth (prospective preferences); and an item asking whether current pregnancies or recent births were wanted, mistimed or unwanted (retrospective desires). Many studies have demonstrated the relatively high validity and reliability of the prospective preferences item, including its predictive validity [12–15], and therefore the now-standard survey-based indicator of unmet need for contraception relies on this item [16]. But the standard indicator of unmet need also relies on the retrospective report of whether current pregnancies and recent births were wanted and on time, and empirical evidence raises serious questions about the validity and reliability of this item [17–19]. Many scholars have suggested that fertility desires are multidimensional and variable [20, 21]. This stance has been bolstered by recent research which highlights nuance, tentativeness, and fluidity in fertility desires [22–24]. Empirical confirmation of the latter is the documented fact that these desires are subject to change over the life course [17, 25]. All this has led to calls for improving the standard survey measurement of fertility desires, with the aim of providing a foundation for more valid and reliable estimation of both unmet need for family planning and rates of unintended pregnancy.

For the last two decades, most research on the causes of unmet need have made use of data collected under the Demographic and Health Surveys (DHS) Program. Studies have been of two forms: (i) analysis of social and demographic correlates of current contraceptive use, such as region, education and number of living children; and (ii) analysis of the DHS items on self-reported reasons for non-use. The former body of evidence – studies of social and demographic correlates of unmet need – is certainly informative for policy and programme purposes, as it provides a basis for the targeting of investments and the training of programme personnel. It may also be regarded as suggestive of the more direct causes of unmet need (such as listed above), but this is by implication only and requires an imaginative exercise on the part of the analyst. Studies of social and demographic correlates are no substitute for explicit investigation of the direct causes of unmet need.

Analysis of the second form uses responses to the question which asks all women who wish to stop or wait childbearing but were not using contraception their reasons for non-use. The phrase has changed slightly over years, but the general form of the question is “you have said you do not want (a/another) child soon (or any (more) children). Can you tell me why you are not using a method to prevent pregnancy?” Using these self-reported reasons, studies in the 1990s identified lack of knowledge, fear of side effects and social disapproval by family as the major obstacles to contraceptive use in many countries that participated in the first round of DHS [26], and lack of information, opposition to contraception, ambivalence about future childbearing emerged as main causes of unmet need in the second DHS round [27]. More recently, Sedgh and Hussain assessed women’s direct responses on reasons for non-use using recent DHSs from 51 developing countries, and showed fear of side effects/adverse health risk and infrequent sex as the dominant reasons for contraceptive non-use [28]. In this analysis, lack of access or information was not a dominant reason for non-use of contraception except in countries in Middle and Western African countries. Opposition by women themselves, husbands or relatives were commonly cited among women in Southern Asia and Western Africa, but not elsewhere.

These self-reported subjective rationales for non-use of contraception are, however, unlikely to capture a complex, diverse and competing sets of reasons for unmet need, and interpretations of the responses require caution [11, 27]. A study in Ghana suggested that informational access measured by knowledge of two popular modern methods and supply sources contributes to unmet need more than was previously considered from the self-reported reasons [29]. Moreover, there is lack of comparable information from contraceptive users. For instance, side effects and health concerns are the dominant self-reported reasons for non-use. However, in the absence of information on whether side effects and health concerns are equally common among users, it is impossible to be confident that these two interrelated factors truly distinguish non-users from users.

Furthermore, unmet need for family planning by definition takes no account of whether individuals or couples use appropriate and suitable methods based on their fertility preferences, sexual behaviours including coital frequency, and their health [30]. Increasing proportions of women who have unmet need in low- and middle-income countries are past contraceptive users [31]. Little is known about the extent whether these women discontinued a particular method, or discontinued practice of contraception altogether [32]. If dissatisfaction to a specific method is a reason for discontinuation, family planning programmes need to refine their focus on responding to women’s demand for more suitable satisfactory methods. Therefore, there is a clear gap in scientific evidence on understanding of generic and method-specific attitudes and experiences of contraceptive use to meet the reproductive health needs of women.

## Aim and objectives

The overarching aim of this multi-site cohort study is to investigate the reasons for unmet need for family planning, with particular attention to measurement of fertility preferences in rural and urban Kenya, and in rural Bangladesh. We propose a conceptual framework of reasons for unmet need for family planning to measure hypothesized causal factors for users as well as non-users, including past users. Our analysis will reveal the statistical power of factors to discriminate between users and non-users and infer causation of unmet need.

The specific objectives at the baseline are:

1. To assess whether a few additions to standard DHS questions on future fertility preferences add significant explanatory power to the probability of current use and intended use for those not currently at risk of getting pregnant.
2. To assess the extent to which hypothesized causal factors (generic concerns about contraception and method-specific attributes) are significantly associated with current use, future intended use and non-use.
3. To assess the relationship between method-specific perceptions and past contraceptive experiences, and method-specific use, intention to use and non-use.

With two rounds of follow-up interviews, additional objectives are:

4. To measure the validity of enhanced prospective fertility preference data in terms of their power to predict subsequent pregnancy and births.
5. To measure the validity of enhanced prospective fertility preference and other possible factors on contraceptive perceptions and experience to predict contraceptive use-continuation, adoption and unmet need for family planning.
6. To assess consistency of prospective fertility intentions and retrospective statements about intendedness.

To achieve these objectives, we developed a new instrument to gather extensive information on women’s perceptions of contraception (generic and method-specific) and their past contraceptive experience. This instrument will allow for more complexity in fertility preferences than is standard in demographic surveys. The newly developed instrument can be used prospectively in health demographic and surveillance systems (HDSSs) or cross-sectional studies.

The study will be carried out by London School of Hygiene and Tropical Medicine (LSHTM), Population Council, African Population and Health Research Center (APHRC) and icddr,b, in collaboration with Ohio State University, under the Strengthening Evidence for Programming on Unintended Pregnancy (STEP UP) Research Programme Consortium.

## Methods/design

### Causal Framework for reasons for unmet need for family planning

**Fig. 1:**
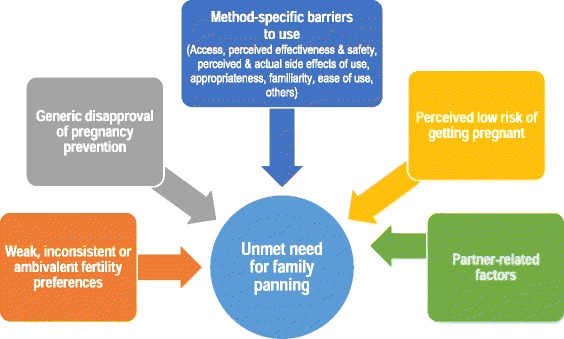
Causal framework for the reasons for unmet need for family planning

Despite the effort to investigate reasons for unmet need for family planning in the 1990s, no convincing causal framework has been proposed. Largely based on the list of factors identified in the study by Casterline, Perez and Biddlecom [5], literature review in the 1998 United Nations’ report [30], and discussions at an Expert Meeting on *Conceptualizing and Measuring Unintended Pregnancy and Birth: Moving the Field Forward* that was held in 2015 [33], we developed a causal framework for unmet need for family planning as shown in Fig. 1.

1. **Weak or inconsistent or ambivalent fertility preferences**
  Ambivalent, weakly held or vacillating fertility preferences are likely to be a major cause of non-use and apparent unmet need for family planning. The extent of attachment to stated preferences are often unknown and weak commitment to the stated prospective desire may be a key underlying contributor to unmet need [5, 6, 27, 34]. Insufficient motivation to avoid pregnancy prevents women to overcome even minimal barriers to practicing contraception [30]. Stated fertility preferences may not be fixed, and they may be inconsistent, unstable or contradictory [35]. Conventional cross-sectional surveys including DHSs do not capture these features. In contrast, a prospective cohort design allows us to assess an effect of fertility preferences on current and future contraceptive use and non-use, and examine stability of fertility desires over time.
   Weak attachment to stated preferences is likely to be more common for women who want to delay childbearing rather than those wanting to stop altogether [27]. While increasing numbers of women desire to stop childbearing, it is important to note that birth spacing is a dominant motivation to avoid pregnancy in sub-Saharan Africa [36].
2. **Generic disapproval of preventing pregnancy**
  Various social, cultural, psychological, economic barriers prevent couples from adopting and continuing use of contraception even if they are sufficiently motivated to avoid pregnancy [30]. The barriers may be generic disapproval of contraception or may relate to specific methods.
   Generic ambivalence about contraception in general reflects the fact that deliberate pregnancy-prevention within marriage is a radical innovation that concerns a central component of all cultures, namely reproduction. And like all radical innovations, the idea of contraception may provoke initial disquiet or hostility particularly in low use countries. In low use settings, basic lack of informational access (knowledge of method and source of supply) is an important factor [37]. As discussed earlier, other dimensions of access (distance to source, cost, quality of services) do not appear to be very important [7, 38]. To assess the role of geographic access, a widely dispersed sample is needed and such a design is not envisaged. Quality of services may be important for continuation [39], but cannot be assessed as a reason for non-use because by definition non-users have no relevant experience.
3. **Method-specific barriers to use**
  Method-specific factors include: a) information, geographical, financial access and accessibility and ease of obtaining if desired; b) perceived effectiveness in preventing pregnancy; c) perceived safety of short- and long-term use, and perceived fear for future childbearing; d) perceived and actual side effects of use; e) appropriateness for someone like respondent; f) familiarity measured by contraceptive use by respondents’ social network and their experiences; and g) ease of use, including possibility of clandestine use. For past and current users, satisfaction with a method may be important [32].
   We hypothesise that the strongest influence on adoption of a specific method is a woman’s perception of the attitudes of her peer group towards the method, and their uptake and experience of that method [10], though the effects may vary across settings or stage of fertility transition [40]. Concerns about methods typically relate to side-effects, menstrual disruption, health concerns and worries about permanent or long term impairment of future fertility [28, 41–44]. These concerns may be based on actual experience with the method or on the experience of peers or on “rumour” [45]. Fear of side effect is also the major reason for discontinuation [46].
4. **Perceived low risk of getting pregnant**
  Women may not practise contraception because they consider themselves to be at no or low risk of conceiving, no matter what reproductive preferences they bear. This includes permanent factors, such as infertility, and temporary factors such as lactational amenorrhea, sexual abstinence and low coital frequency. Particularly in high contraceptive use settings, perceived infecundity and sub-fecundity is a major reason for non-use [5, 47]. Temporary low risk of pregnancy because of lactational amenorrhea and low coital frequency are also important [28]. Due to increasing short- and long-term migration, low level of, or sporadic engagement in, sexual activities may lead to perceived low risk of pregnancy and hence to inconsistent use of contraception [37, 48, 49].
5. **Partner-related factors**
  Perceived or actual partner’s fertility preferences, attitudes to contraception and communication may influence woman’s decision-making autonomy. Opposition of the partner to contraception in general can be a barrier to use [5, 9]. The study using the DHS data from 24 sub-Saharan African countries showed that fertility preferences were in agreement among most couples [50]. However, when there were substantial differences, couples were less likely to use contraception. A similar result was found in Northern Malawi, where a significant drop in contraceptive use among polygynous couples when the couple’s fertility aspiration differed [51].

### Settings

The study will be carried out in the Nairobi Urban Health and Demographic Surveillance System (NUHDSS) and Homa-Bay in Kenya, and Matlab Health and Demographic Surveillance System in Bangladesh. The Nairobi and Matlab sites were selected because they are long-standing HDSSs which monitor vital events routinely and provide a platform for nested studies. The Homa-Bay site was chosen due to the high level of unmet need for family planning and unintended pregnancy. These sites provide ideal setting to test this newly developped instrument to investigate unmet need for family planning over 18 months.

### Nairobi, Kenya

In Nairobi, this study is being carried out in the NUHDSS. The NUHDSS set up in 2002 by APHRC is located in Korogocho and Viwandani slums, which are 6 to 7 km from Nairobi city centre. The NUHDSS covers 14 villages in both slum settlements. The NUHDSS follows a population of about 65,000 individuals living in about 24,000 households in the two settlements [52]. Although there are marked differences between the slums – Korogocho has a more settled population while Viwandani is home to a young and highly mobile population−, both settlements are characterised by high levels of unemployment, sub-standard and overcrowded housing, limited education and social services, high levels of crime and insecurity and inadequate water and sanitation infrastructure. Households covered by the NUHDSS are visited every 4 months to collect data on key sociodemographic and health measures including births, deaths, migration, immunisation, livelihoods, as well as household amenities and assets. The NUHDSS therefore provides a platform for nested studies investigating the inter-linkages between poverty and health and other outcomes facing slum dwellers.

### Homa-Bay, Kenya

Homa-Bay County is located along the shores of Lake Victoria in western Kenya, and covers an area of approximately 3183 km^2^. In 2009, Homa-Bay County had an estimated population of 963,794 persons. The county was purposefully selected due to its large rural population estimated at 85.7% [53]. The population is rapidly growing and was projected to rise to approximately 1.18 million by 2017. This growth is largely attributed to high fertility, which is currently estimated at 5.2 children per woman, compared to a national average of 3.9 children per woman [54]. The level of contraceptive use among currently married women aged 15–49 years is modest at 47% while unmet need for family planning is among the highest in the country at about 26% [54]. Furthermore, Homa-Bay County has a perpetual burden of high unintended pregnancy and the highest HIV prevalence estimated at 27% in 2012 [55].

### Matlab, Bangladesh

Matlab is a rural area located about 55 km south-east of the capital city of Dhaka. Since 1966, icddr,b has been maintaining a HDSS in Matlab that is the longest and largest surveillance site in the developing world. Matlab is divided into two parts; icddr,b service area and government service area. The surveillance system records births, deaths, migration, marriages and divorces of household members in both areas that were collected by the village-based Community Health Research Workers (CHRWs). The monitoring system covers 220,000 residents in 142 villages of the HDSS area. Data are collected only from individuals who are regular residents. A resident is a person residing in the HDSS area permanently or continuously for at least six months.

Although the difference between the two areas has narrowed, contraceptive prevalence is higher in icddr,b service area (54%) than in the government service area (42%) [56]. Yet, the prevalence is lower than the national average (62%). Nearly 40% of users in the iccdr, b service area use injectables and 32% were pill users. In contrast, pill use is more common (44%) than injectables (27%) in the government service area.

### Study design

The study will be a prospective cohort study and we plan to follow up women to monitor reproductive outcomes and adoption and continuation of contraceptive use over 18 months in three study settings in two countries, Kenya and Bangladesh. Because cohort studies allow for repeated collection of data from the same individual over time, we will be able to assess the predictive power of baseline measures on reproductive outcomes, contraceptive adoption and continuation of use and accurately observe and measure changes. The data at baseline will be compared with data at 12 and 18 months of follow-up, to determine the overall temporal trends and dynamics in fertility preferences and pregnancy outcomes. A questionnaire will be administered through face-to-face interviews by trained fieldworkers in the respective local languages.

Two thousand six hundred married or cohabiting women aged 15–39 years living in each of the three study areas will be recruited. The upper age limit is based on the need to recruit women who are likely to become pregnant during subsequent rounds. Their husbands/partners will be excluded because matched couple data analysis is beyond the scope of this study. In addition, the benefits from including men has been small in previous studies due to difficulty in recruiting men.

### Tool development

We reviewed existing literature and more than 30 instruments on fertility preferences and reasons for non-use of contraception conducted in low-, middle-, and high-income countries, and compiled question items by themes. This includes instruments from the DHS, the Determinants of Unintended Pregnancy Risk study in New Orleans, the US- based National Survey of Family Growth, and the Fog Zone study by the Guttmacher Institute. Subsequently, a new questionnaire was developed using the compilation of the question items. A draft instrument was reviewed through consultative process with dozens of experts in the field.

The questionnaire has three main components: a sub-set of DHS items measuring socio-demographic characteristics, reproductive history, and sexual activity; additional questions on prospective and retrospective fertility preferences designed to capture ambivalence and uncertainty; and two large blocks of items on (i) generic concerns about contraception and (ii) method-specific attributes. The method-specific items encompass eight modern and traditional methods. A list of selected question items is presented in Table 1.

**Table 1:** List of selected question items

Category	Question items
Background characteristics of women and husbands/partners	Age, level of education, current marital status and history, occupation including casual work, religion, co-residential status with husband/partner, ethnicity (only in Kenya), perceived risk of HIV infection (only Homa-Bay)
Reproduction	Parity, number of living children, age of the last child, current pregnancy status, duration of pregnancy, outcome of pregnancy at round 2 and 3 (live births, survival status, miscarriages, abortions)
Past, current and future contraceptive use	Knowledge of contraceptive methods, current use (month-to-month use at round 2 and 3), use of emergency contraception, reasons for non-use, intention for future use, preferred method, intention to switch a method
(1) Fertility preferences	
Prospective fertility preferences	Future desire for children, preferences for timing of pregnancy, importance of avoiding pregnancy, potential changes in fertility preferences, feelings about getting pregnant
Retrospective fertility preferences	Pregnancy wantedness, preferences for timing of pregnancy, importance of avoiding pregnancy, use of family planning before pregnancy, feelings about becoming pregnant
Ideal number of children	Ideal number of children
(2) Generic disapproval of pregnancy prevention	Approval of/opposition to contraceptive use, importance of features that determine method-choice
(3) Method-specific barriers to use	Familiarity, access, perceived effectiveness, safety, side effects, ease of use, appropriateness of someone like respondent, partner-related factors, satisfaction
(4) Perceived risk of getting pregnant	Perceived infecundity, frequency of sexual activity, postpartum insusceptibility, knowledge of safe period during breastfeeding
(5) Partner-related factors	Perceived partner’s fertility preferences, partner’s opposition to contraception

### Sample size

The main outcomes are intended use, adoption and continuation of contraceptive use, and pregnancy or birth. Although we will explore exposures and predictors in relation to fertility intentions and general and method-specific attitudes, we can assume both exposure/predictor and outcome variables to be dichotomous. For instance, there may be 20% in the unexposed and 40% in the exposed positive on outcome variable, such as current contraceptive use. Our sample size calculation is based on the following formulae for comparing two proportions to be able to detect a 30% differences in two proportions at 95% confidence level and 80% power [57].

$$ {n}^{\prime }=\frac{{\left({Z}_{\alpha /2}\sqrt{2\overline{P}\overline{Q}}+{Z}_{\beta}\sqrt{P_1{Q}_1+{P}_2{Q}_2}\right)}^2}{{\left({P}_2-{P}_1\right)}^2} $$

Where:

- ${\mathrm{n}}^{\prime }$ is the required sample size of the individuals of target population;
- $\mathrm{P}$ is the proportion of outcome in exposed and unexposed groups;
- $$ \mathrm{Q} $$ is 1-P;
- z_α/2_ is significance level (for a two-tailed test, z _α/2_ is equal to 1.96 if significance level is 5%)
- z_β_ is one-sided percentage point of the normal distribution corresponding to 100% - the power (at 80% power, z _β_ is 1.84).

As the distribution of exposure is unknown, different ratios of sample size of the exposed to the unexposed (20% vs 80%, 30% vs70%… 80% vs 20%) were used to calculate sample sizes. The continuity correction factor is applied to the normal approximation of the discrete distribution. Ten percent of non-response is assumed, but this needs to be adjusted for each site. Furthermore, the primary interest in the single round survey is women who are in need for family planning, i.e., women who are not currently pregnant, are not in postpartum amenorrhea, and do not want a child soon. Based on the latest DHS surveys in the three countries, it is estimated that these women account for about 50% of women in union aged 15–39. Therefore, required sample sizes for women in need for contraception is the doubled size for the overall sample size, leading to as 2567 women (Table 2).

**Table 2:** Sample size calculations for the single round survey

Significance level	Power	% of outcome in unexposed	Effect size (relative risk)	Sample size for women in need for FP	Overall sample size
0.05	80%	30%	30% (1.3)	1283	2567

The sample sizes were also calculated for the prospective study using the same formulae and assumptions used in the single round survey. Based on the previous studies, it is estimated that risk ratio of pregnancy between women wanting a child soon and those wanting to wait for 5 or more years or wanting no more children rate is 1.3 or higher. The non-response and attrition rates are adjusted for each site (Table 3).

**Table 3:** Sample size calculation for the prospective survey

Significance level	Power	% of outcome in unexposed	Effect size (relative risk)	Attrition	Sample size for women at risk of unintended pregnancy at baseline	Overall sample size
0.05	80%	30%	30% (0.7)	45%	1998	2597

According to the above calculations, we arrived at 2600 women in union aged 15–39 to be able to detect at least a 30% difference at 80% of power both in single round and prospective surveys.

## Sampling method

### Nairobi, Kenya

Using the NUHDSS database, a listing of all women aged between 15 and 39 years who are formal residents will be generated with identifying information including name, age, sex, and location and structure numbers. This listing will form the study population. Married or co-habiting participants between the ages of 15 and 39 years will then be randomly selected from households in the HDSS database. For each study participant, a new non-identifying unique ID will be generated and assigned to ensure anonymity. As of May 2016, about 14,867 women aged 15 to 39 years from 25,243 households were present in the demographic surveillance area in the most recent survey round. Of these, 5835 were women resident in 9181 households in Korogocho, and 9032 were women resident in 16,062 households in Viwandani.

### Homa-Bay, Kenya

A representative sample of 3120 married or cohabiting women aged 15–39 years living in three purposely selected sub-counties in Homa-Bay County will be drawn for the survey. A two‐stage cluster sampling design will be used for the Homa-Bay study. Stage one will involve selecting a random sample of clusters (sub-locations) from three purposely selected sub-counties— Rachuonyo North, Rachuonyo South and Ndhiwa. A total of 12 rural clusters will be sampled with equal probability independently within the selected sub-counties. We will then conduct a household listing in all sampled sub-locations, which will form the sampling frame for the second stage. The following information will be captured using a household listing form; names of all members starting with head of household, relationship of each member to the household head, sex, age, and marital status of each member.

Married or co-habiting participants between the ages of 15 and 39 years will then be randomly selected from the household list. A systematic random sampling method will be adopted as it allows even distribution of the sample across the clusters and yields good estimates for the population parameters. Sampling without replacement of eligible women will be done at the office and assigned to the enumerators. Approximately, 1040 eligible women will be sampled from each sub-county, and interviewed using the questionnaire.

### Matlab, Bangladesh

According to Matlab HDSS database (as of December 2015), about 34,308 women are currently married and aged 15 to 39 years. Among them, 18,212 women resides in icddr,b service area and 16,096 women resides in government service area. Each service area is divided into a number of blocks (units), four blocks in icddr,b service area and three blocks in government service area. From both icddr,b and government sites, a total of 3000 women will be selected by simple random sampling, and out of them 2600 respondents will be interviewed after obtaining informed written consent. Participants will be replaced if age and marital status does not match.

In each site, all attempts will be made to locate the selected women for interviews. Call back schedule will be made when any woman is not found in her location. If the woman is not found after three attempts or visits during study period, she will be considered lost to follow up for that particular round of interview and will not be replaced.

### Data management and quality control

Data collection will be carried out on digital Open Data Kit (ODK), an open source data management software, in the two Kenyan sites. The two sites will collaborate in programming the questionnaire in ODK. Research assistants will be trained to use a tablet for administering the questionnaire. These applications allow for paperless and prompt data collection, transmission, verification and storage. In Matlab, paper-based questionnaire will be administered by trained fieldworkers, and data entry and cleaning will be conducted by trained researchers in icddr,b, Dhaka.

Local supervisors will check completed interviews at the end of each day during the data collection period. They will monitor and conduct random check to ensure quality control and interviewers’ adherence with confidentiality procedures. In addition, data quality will be simultaneously monitored by data analysts.

Data will be cleaned and entered into a purpose-designed database in each office. After entry, data cleaning will be carried out. The cleaned data will be stored on a computer drive with restricted access. This will be transferred into Stata for storage and analysis. Hard copies of the questionnaires will be kept and archived in accordance with the institutional data regulatory guidelines.

### Ethical considerations, training and quality control

Ethical approvals for this study were obtained from the Institutional Review Boards of the London School of Hygiene and Tropical Medicine and Population Council as well as by the AMREF Ethics and Scientific Review Committee for the Nairobi site, Kenyatta National Hospital-University of Nairobi Ethics and Research Committee for the Homa-Bay site and icddr,b Institutional Review Board (Research Review Committee and Ethical Review Committee) for the Matlab site, respectively.

Informed consent will be obtained separately for each interview through use of the informed consent forms. All married and co-habiting adolescents aged 15 – 17 years will be considered emancipated minors for which parental permission is not required. All respondents will be informed about the objectives, procedures, benefits, and risks of the study through the process of obtaining informed consent.

There is potential risk that a woman who decided to participate in the study may feel discomfort or distress when being asked a number of questions on potentially sensitive subjects including experiences with various pregnancy outcomes. It is possible that women who experience out-of-wedlock pregnancies or certain pregnancy outcomes, such as abortion, may be stigmatised or depressed. To minimise these risks, careful steps has taken in the questionnaire design. The English questionnaire will be translated into and back translated from Swahili for Nairobi, Dholuo for Homa-Bay, and Bengali for Matlab.

The selection, characteristics and training of research assistants will also be a key step in minimising the risk of distress that might be posed to participants. Research assistants will also be trained on the study design and procedures as well as ethical considerations in the research during approximately one-week training specifically for this study. Training also includes field-testing the tool among a small group of women with similar characteristics as the study population to identify potentially negative consequences and will be modified accordingly. Research assistants will be trained to listen intently without displaying any judgmental attitude towards information they receive from the informants. They will be instructed to stop the interview abruptly if the informant is upset and refer those who need psychosocial support to specific services. Interviews will be conducted in private after obtaining written informed consent during times and at venues that are convenient to participants. It will further be made clear to participants during the consent process that they have the right to withdraw from the research at any time without reprisal. Participants who show signs of distress will be referred to the nearest facilities for appropriate care.

## Discussion

Policy and programmes intended to reduce unmet need in low- and middle-income countries, particularly efforts towards goals set for FP2020 and the Sustainable Development Goals (SDGs), should be informed by clear understanding of the causes of unmet need for family planning to better reflect the population needs and to more effectively target planning and investments. To this end, this study will field an innovative instrument in three sites in Kenya and Bangladesh. The information to be collected will support a rigorous assessment of reasons for unmet need for family planning.
